# Associations between Dietary Intake of Choline and Betaine and Lung Cancer Risk

**DOI:** 10.1371/journal.pone.0054561

**Published:** 2013-02-01

**Authors:** Jun Ying, Mohammad H. Rahbar, D. Michael Hallman, Ladia M. Hernandez, Margret R. Spitz, Michele R. Forman, Olga Y. Gorlova

**Affiliations:** 1 Department of Epidemiology, University of Texas MD Anderson Cancer Center, Houston, Texas, United States of America; 2 Department of Epidemiology, University of Texas School of Public Health, Houston, Texas, United States of America; 3 Department of Nutritional Sciences, Dell Pediatric Research Institute, College of Natural Sciences, University of Texas – Austin, Austin, Texas, United States of America; 4 Duncan Cancer Center, Baylor College of Medicine, Houston, Texas, United States of America; Virginia Commenwealth University, United States of America

## Abstract

Evidence from human and animal research indicates that choline metabolic pathways may be activated during a variety of diseases, including cancer. We report results of a case-control study of 2821 lung cancer cases and 2923 controls that assessed associations of choline and betaine dietary intakes with lung cancer. Using multivariable logistic regression analyses, we report a significant association between higher betaine intake and lower lung cancer risk that varied by smoking status. Specifically, no significant association was observed between betaine intake and lung cancer among never-smokers. However, higher betaine intake was significantly associated with reduced lung cancer risk among smokers, and the protective effect was more evident among current than former smokers: for former and current smokers, the ORs (95% CI) of lung cancer for individuals with highest as compared to lowest quartiles of intake were 0.70(0.55–0.88) and 0.51(0.39–0.66) respectively. Significant linear trend of higher betaine intake and lower lung cancer risk was observed among both former (p_trend_ = 0.002) and current (p_trend_<0.0001) smokers. A similar protective effect was also observed with choline intake both in overall analysis as well as among current smokers, with p-values for chi-square tests being 0.001 and 0.004 respectively, but the effect was less evident, as no linear trend was observed. Our results suggest that choline and betaine intake, especially higher betaine intake, may be protective against lung cancer through mitigating the adverse effect of smoking.

## Introduction

**Figure 1:**
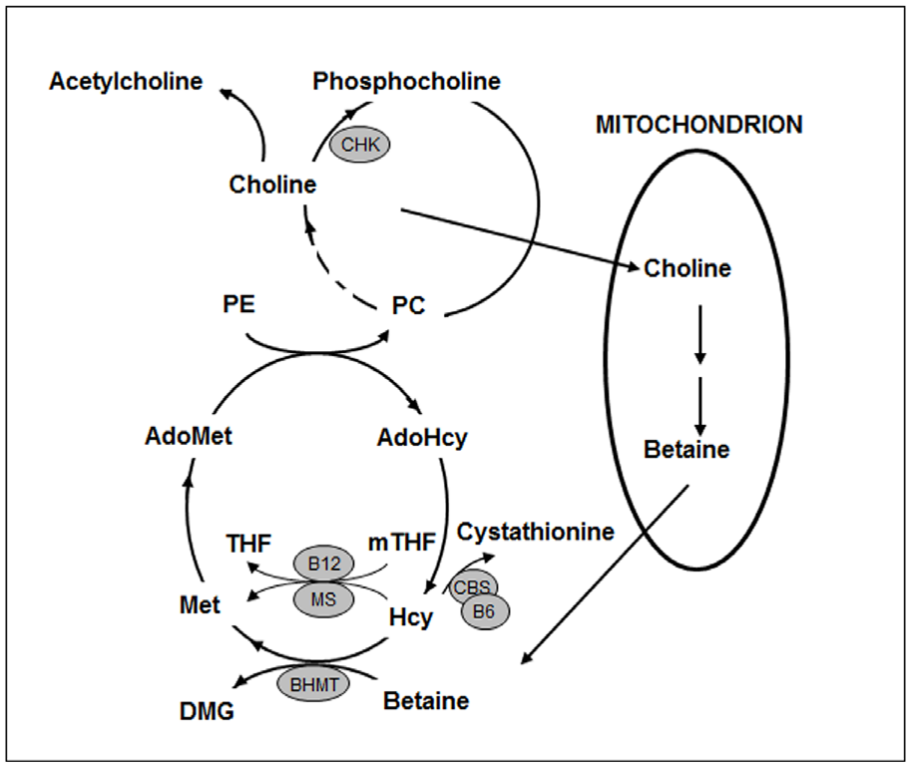
Choline and Betaine Metabolism. Abbreviations: PC – phosphatidylcholine; PE – phosphatidylethanolamine; AdoMet – S-adenosylmethionine; AdoHcy – S-adenosylhomocysteine; THF – tetrahydrofolate; mTHF –5-methyltetrahydrofolate; Met – methionine; Hcy – homocysteine; DMG – dimethylglycine; MS – methionine synthase; BHMT – betaine-homocysteine S-methyltransferase; CBS – cystathionine β-synthase; B6– vitamin B6; B12– vitamin B12.

A locus on chr15q25 that includes a cluster of nicotinic acetylcholine receptor subunit genes CHRNA3, CHRNB4 and CHRNA5 was identified to be robustly associated with lung cancer risk in genome wide association studies, suggesting the potential relevance of choline and its metabolites in lung cancer risk [1], [2]. Choline and betaine are nutrients that can be either obtained from the diet or synthesized metabolically. Choline, the precursor of betaine, is abundantly present in egg yolks, beef, chicken, liver and soybeans [3], and is converted to betaine through a two-step oxidation process that occurs in the mitochondria of liver and kidney. Most of the remaining choline is phosphorylated to phosphocholine by choline kinase (CHK) and is further converted to phosphatidylcholine (PC) (Figure 1). Choline is important for cell membrane structure as well as neurotransmission; is associated with neurodevelopment and hippocampus cell proliferation, differentiation, and apoptosis in animals; and may affect brain development and cognitive functions in humans [4].

Betaine is a nutrient abundant in animal foods, especially seafood, and plant foods such as wheat bran and spinach [3], [5]. It is a methyl donor for the betaine-homocysteine methyltransferase (BHMT)-mediated reaction that converts homocysteine (Hcy) to methionine (Met) [6]. Betaine serves as an osmolyte that regulates cell volume and protects cells and proteins from environmental stresses such as ionic stress and elevated temperature [7]. It is involved in liver metabolism [8], [9].

Choline, betaine, methionine, and folate are major methyl donors in humans. Deprivation of methyl donors causes DNA hypomethylation. Global DNA hypomethylation, regional hypermethylation of tumor suppressor genes and regional hypomethylation of oncogenes and prometastatic genes are associated with nearly all types of cancers [10]–[15]. Many studies have revealed associations between choline/betaine metabolism and cancer [16]–[19], while others have examined the role of dietary intakes of choline and betaine and risk for different cancers, such as breast, colorectal, epithelial ovarian and liver cancers [20]–[24]. To our knowledge, no study has reported on the association between betaine or choline intake and lung cancer. Lung cancer is the second most common cancer, and the leading cause of cancer death, in both men and women in the United States, and there is an urgent need for promising dietary preventive strategies to reduce lung cancer risk, especially amongst smokers. In the current study, we explore the association between lung cancer risk and dietary intake of choline and betaine.

## Materials and Methods

### Study Subjects

This study is part of a previously described molecular epidemiological case-control study carried out by The University of Texas MD Anderson Cancer Center (UTMDACC) that aims to assess susceptibility markers for lung cancer [25], [26]. Briefly, patients with newly diagnosed, histologically confirmed lung cancer were recruited prior to initiation of radiotherapy or chemotherapy at UTMDACC between July 1995 and February 2009. Controls without a previous diagnosis of cancer (except for non-melanoma skin cancer) were recruited from the Kelsey-Seybold Clinics, Houston’s largest private multidisciplinary physician group. There were no age, sex, race/ethnicity or cancer stage restrictions, but the study attempted to match numbers of cases and controls within each smoking status, and age within 5 years of difference between cases and controls. A total of 5942 individuals were included in the analysis. The study was approved by the UTMDACC Institutional Review Board.

### Data Collection

A 45-minute personal interview was conducted by a trained UTMDACC research interviewer for each newly recruited subject, using a structured questionnaire. Information on socio-demographic characteristics, smoking and second-hand smoke (SHS) exposure history, prior respiratory conditions and exposures, medical history, and family history of cancer was obtained during the interview. Never-smokers were defined as individuals who smoked less than 100 cigarettes in their lifetime. Former smokers were defined as individuals who had quit smoking at least one year prior to diagnosis (cases) or the interview (controls). Individuals who reported currently smoking or had quit smoking less than one year prior to enrollment were classified as current smokers. Individuals with daily cigarette consumption of at least one pack were considered heavy smokers. Family cancer history was based on a cancer diagnosis in at least one first-degree family member. Dust exposure represents the presence of any dust-related exposure including exposure to sawdust, coal dust, sand, metal and cotton. SHS exposure was defined as exposure to any type of second-hand-smoke (at work, at home, or during leisure time away from home or work). A nicotine addiction score (0–10) was calculated using a modified Fagerstrom Test of Nicotine Dependence (FTND) and used as a measure of nicotine dependency [25].

Dietary data were collected using a modified version of the Health Habits and History Questionnaire Food Frequency Questionnaire (FFQ) developed by the National Cancer Institute. The validity and reliability of this questionnaire has been documented [27], [28]. The FFQ includes a semi-quantitative list of 214 food and beverage items including ethnic foods popular in the Houston community, as well as questions about vitamin and mineral supplement use, eating at restaurants, and food preparation methods. The questionnaire assessed usual food intake during the year prior to enrollment in the study for controls and during the year prior to diagnosis for cases. Nutrient intake was calculated using the DIETSYS+Plus version 5.9 dietary analysis program (Block Dietary Data Systems, Berkeley, CA) to calculate grams of intake per day.

The USDA Database for the Choline Content of Common Foods, Release 2 [29] was used to obtain the choline and betaine composition of individual foods. Source of total calories and alcohol values for food and beverage items were Standard Reference, Release 21 [30]. We calculated average daily intake of choline and betaine by multiplying the frequency of consumption of each food item by its choline and betaine content, and a summary measure for all foods was generated for choline and betaine. Total choline intake was calculated as the sum of choline intake from free choline, phosphatidylcholine, phosphocholine, glycerophosphocholine and sphingomyelin.

### Statistical Analysis

The initial dataset included 2925 lung cancer cases and 3017 controls. Total caloric intake ranged from 5 to 10965 kcal/day. Individuals with total caloric intake below the 1^st^ and above the 99^th^ percentiles for each sex were considered as outliers and were excluded from the analysis. The total caloric intake in the remaining sample ranged 749–5939 kcal/day for males, and 593–5522 kcal/day for females, with 181 individuals being excluded. Nineteen individuals (0.3%) were excluded due to missing data on race/ethnicity or for being other than African American, Hispanic, or non-Hispanic White.

Dietary intake of choline and betaine was adjusted by total calories using the residual method proposed by Willett *et al.* [31]. Energy-adjusted choline and betaine intake were categorized into quartiles based on their distributions in controls. Logistic regression was used to evaluate associations between choline and betaine intake and lung cancer. Based on previous studies [26], [32]–[35], age, sex, race/ethnicity, smoking status, dust exposure, previously diagnosed respiratory diseases (hay fever, asthma, and emphysema), family cancer history, SHS in never-smokers, nicotine dependency (addiction index) and cumulative tobacco exposure (smoking pack-year) among smokers, time since smoking cessation for former smokers, as well as alcohol consumption and total energy intake were regarded as potential confounders for the association between choline and betaine intake and lung cancer. All potential relevant confounders were tested for association with choline and betaine intake as well as with lung cancer risk. Variables associated with both choline or betaine intake and lung cancer risk at significance level of p<0.25, that also changed the odds ratio for crude association between choline or betaine intake and lung cancer by ≥10%, were considered as confounders [23]. Age, sex, race/ethnicity and smoking status are known confounders and were included in multivariable regression models along with total caloric intake, for analyses of the whole dataset, and age, sex, race/ethnicity and total caloric intake were included in regression models stratified by smoking status. Tests for linear trends were assessed by applying Wald tests to ordinal values of corresponding variables, entered as continuous variables.

Interactions between each covariate (other than smoking status) and choline or betaine intake were tested individually using likelihood ratio (LR) tests. Variables that were neither confounders nor effect modifiers were excluded from the model.

To ensure that no confounders were eliminated due to our stringent model selection criteria, complete multivariable regression models contained all individually evaluated potential confounders and risk factors as listed in previous text were tested and compared with above reduced models.

For all binary variables, a third category was added to denote missing observations. Among all continuous variables, only three variables contained missing data, and the maximal missingness was 2%. Therefore, missing data were ignored during analyses. Zero was assigned to smoking related variables pack-year of smoking and addition index, for never smokers. Current age was assigned to variable time since smoking cessation for never smokers, and 0 was assigned to current smokers for the variable.

All data were analyzed using SAS Version 9.2 (SAS Inc., Cary, NC).

## Results

After data cleaning and processing, 5744 of 5942 individuals remained for analysis, which included 2821 cases and 2923 controls. Demographic characteristics of the sample and summary statistics for potential confounders are shown in Table 1. There were significant differences (p<0.05) between cases and controls for all variables listed except total calories and alcohol consumption. All variables in Table 1 were significantly (p<0.05) associated with betaine intake, while race/ethnicity, sex, smoking status, dust exposure, addiction index and alcohol intake were significantly associated with choline intake (data not shown).

**Table 1:** Comparison of demographic and other characteristics between cases and controls (n = 5744).

Variable		Cases (n = 2821)		Controls (n = 2923)		P*
		No.	%	No.	%	
Sex	Male	1507	53.42	1448	49.54	0.003
	Female	1314	46.58	1475	50.46	
Race	Black	389	13.79	383	13.10	0.028
	Hispanic	154	5.46	209	7.15	
	White	2278	80.75	2331	79.74	
Smoking status	Never	453	16.06	693	23.71	<0.0001
	Former	1225	43.42	1168	39.96	
	Current	1143	40.52	1062	36.33	
Emphysema	Yes	524	18.57	169	5.78	<0.0001
	No	2050	72.67	2571	87.98	
Asthma	Yes	386	13.68	351	12.01	<0.0001
	No	2176	77.14	2407	82.35	
Hay fever	Yes	421	14.92	621	21.25	<0.0001
	No	2127	75.40	2142	73.28	
Family history^a^	Yes	1637	58.03	1545	52.86	<0.0001
	No	757	26.83	1062	36.33	
Dust exposure^b^	Yes	818	29.00	662	22.65	<0.0001
	No	1569	55.62	1948	66.40	
SHS^c^	Yes	1597	56.61	1746	59.73	<0.0001
	No	168	5.96	348	11.91	
		No.	Mean(SD)	No.	Mean(SD)	p**
Age (year)		2821	62.49 (10.86)	2923	58.75 (11.47)	<0.0001
Addction index (0–10)^d^		2294	4.56 (2.41)	2200	4.00 (2.59)	<0.0001
Smoking pack-year^d^		2329	49.80 (37.41)	2205	38.78 (29.92)	<0.0001
Time since smoking cessation(year)^e^		1214	14.86 (11.11)	1155	15.95 (11.89)	0.021
Calories (kcal/day)		2821	2204 (907)	2923	2206 (900)	0.936
Alcohol (g/day)		2821	8.24 (18.00)	2923	7.71 (17.88)	0.261

a Total 12.9% missing. ^b^ Total 13% missing. ^c^ Total 32.8% missing. ^d^ Smokers only. ^e^ Former smokers only.

* p-value, two-sided chi-square test. ** p-value, Student’s t-test.

We found significant linear associations between smoking status and total caloric intake, as well as choline and betaine intake (Table 2). Higher total calorie intake was observed with elevated smoking status, where the mean caloric intake increased from 2032 kcal/day among never smokers, to 2337 kcal/day among current smokers (p_trend_ <0.0001). Similarly, energy-adjusted choline intake was the lowest among never smokers (mean = 298.3 mg/day) and highest among current smokers (mean = 305.7 mg/day, p_trend_ = 0.038). Conversely, a significant decreasing trend was observed for betaine intake with increased smoking status, where never-smokers had the highest mean betaine intake (55.7 mg/day), and current smokers had the lowest mean intake (42.7 mg/day, p_trend_ <0.0001).

**Table 2:** Summary Statistics for Energy-adjusted intake by smoking status.

Variable	Statistics	Smoking Status			p*_trend_
		Never	Former	Current	
Total Calories	Mean	2031.5	2166.4	2337.4	<0.0001
(kcal/day)	Median	1856.4	2013.9	2168.4	
	SD	818.9	869.2	961.2	
Choline	Mean	298.3	305.7	305.7	0.038
(mg/day)	Median	290.5	295	298.2	
	SD	72.4	87.4	91.6	
Betaine	Mean	55.7	49.8	42.7	<0.0001
(mg/day)	Median	45.3	39.6	35.8	
	SD	35.4	36.4	30.8	

* p-value, test for linear trend.

Pack-years of smoking was identified as a confounder of the association between betaine intake and lung cancer. Our reduced model for betaine intake therefore adjusted for age, sex, race/ethnicity, total caloric intake, pack-years of smoking and smoking status. Under this model, a significant inverse association was observed between betaine intake and lung cancer in the overall analysis, where individuals of different smoking status were combined (p_overall_ <0.0001, Table 3). Relative to those in the lowest quartile of betaine intake, individuals in the highest quartile of betaine intake were 33% less likely to be lung cancer cases (OR = 0.67, 95% CI: 0.58–0.79). Odds ratios for those in second and third quartiles of adjusted betaine intake, relative to those in the lowest quartile, were 0.83 (95% CI: 0.71–0.96) and 0.78 (95% CI: 0.67–0.90), respectively. Trend test suggested a significant linear trend between increased betaine intake and decreased lung cancer risk (p_trend_ <0.0001). No effect modification was identified for the association between betaine intake and lung cancer. The complete model did not change the association (p_overall = _0.0006, p_trend_ <0.0001). Analyses using reduced models show that, when stratified by smoking status, there was no significant association between betaine intake and lung cancer risk among never-smokers in either individual quartiles or overall statistics (p_overall_ = 0.635). Nor was a linear trend identified (p_trend_ = 0.702). However, significant associations were observed among both former (p_overall_ = 0.023) and current smokers (p_overall_ <0.0001), with an inverse trend by betaine intake. Among former smokers, the association was only significant in the highest quartile of betaine intake, where the odds ratio was 0.71 (95% CI: 0.56–0.89). Trend test identified a significant linear trend between increased intake and reduced OR (p_trend_ = 0.002). The association between betaine intake and lung cancer was most evident among current smokers, where significant associations were observed in all upper quartiles relative to the lowest quartile. For the 2^nd^ quartile, OR = 0.70, 95% CI: 0.55–0.88; for the 3^rd^ quartile, OR = 0.75, 95% CI: 0.59–0.95; and for the highest quartile, OR = 0.51, 95% CI: 0.39–0.66, with a significant linear trend, p_trend_ <0.0001. Similar results were observed when analyses were performed using complete models.

**Table 3:** Association between quartiles of betaine intake and lung cancer risk.

Models		Quartiles of betaine intake				P**_trend_
		1	2	3	4	
	**Case n (%)**	911 (55.51)	713 (49.38)	650 (47.07)	547 (42.80)	
	**Control n(%)**	730 (44.49)	731 (50.62)	731 (52.93)	731 (57.20)	
**Overall**						
Reduced^a^	OR (95% CI)	1.00	0.83 (0.71–0.96)	0.78 (0.67–0.90)	0.67 (0.58–0.79)	<0.0001
	P_overall_	<0.0001				
Complete^b^	OR (95% CI)	1.00	0.85 (0.73–1.00)	0.81 (0.69–0.95)	0.71 (0.61–0.84)	<0.0001
	P*_overall_	0.0006				
**Stratified by smoking status**						
**Never Smokers**						
Reduced^c^	OR (95% CI)	1.00	1.07 (0.73–1.57)	0.87 (0.59–1.27)	1.00 (0.69–1.45)	0.702
	P*_overall_	0.635				
Complete^d^	OR (95% CI)	1.00	1.06 (0.72–1.57)	0.86 (0.58–1.28)	1.03 (0.70–1.52)	0.869
	P*_overall_	0.614				
**Former Smokers**						
Reduced^c^	OR (95% CI)	1.00	0.92 (0.73–1.16)	0.83 (0.66–1.05)	0.71 (0.56–0.89)	0.002
	P*_overall_	0.023				
Complete^d^	OR (95% CI)	1.00	0.95 (0.74–1.21)	0.86 (0.67–1.11)	0.75 (0.59–0.96)	0.017
	P*_overall_	0.112				
**Current Smokers**						
Reduced^c^	OR (95% CI)	1.00	0.70 (0.55–0.88)	0.75 (0.59–0.95)	0.51 (0.39–0.66)	<0.0001
	P*_overall_	<0.0001				
Complete^d^	OR (95% CI)	1.00	0.73 (0.57–0.93)	0.80 (0.62–1.02)	0.55 (0.42–0.73)	0.0001
	P*_overall_	0.0003				

a Adjusted for sex, race/ethnicity, age, pack-years, total caloric intake, smoking status.

b Adjusted for sex, race/ethnicity, age, pack-years, total caloric intake, family cancer history, dust exposure, second-hand smoke, emphysema, hay fever, asthma, addiction index, alcohol, time since smoking cessation, smking status.

c Adjusted for sex, race/ethnicity, age, pack-years, total caloric intake.

d Adjusted for sex, race/ethnicity, age, pack-years, total caloric intake, family cancer history, dust exposure, second-hand smoke, emphysema, hay fever, asthma, addiction index, alcohol, time since smoking cessation.

* p-value, chi-square test using quartiles of betaine intake as categorical variable.

** p-value, test for linear trend using quartiles of betaine intake as continuous variable.

No confounders apart from sex, race/ethnicity, total caloric intake, smoking status, and age were identified for the association between choline intake and lung cancer, and therefore, only these covariates were included in our final reduced multivariable models for choline. Similar to betaine, no variable was identified as effect modifiers for choline intake. A significant negative association between choline intake and lung cancer was observed for both overall analysis (p_overall_ = 0.001, reduced model; p_overall_ = 0.017, complete model; Table 4) and among current smokers (p_overall_ = 0.004, reduced model; p_overall_ = 0.047, complete model), where choline intake was associated with reduced lung cancer odds. However, no significant association was observed among never- and former smokers. Although significant associations were observed in some individual quartiles, the global test for linear trend was not significant in either the overall analysis (p_trend_ = 0.118, reduced model; p_overall = _0.334, complete model) or any of the smoking categories. The Wald test p-values for trend tests were 0.070, 0.843 and 0.113 respectively for never, former and current smokers with reduced models, and 0.058, 0.299 and 0.260 respectively in complete models. No statistically significant interaction was observed between choline and betaine intake (data not shown).

**Table 4:** Association between quartiles of choline intake and lung cancer risk.

Models		Quartiles of choline intake				p**_trend_
		1	2	3	4	
	**Case n (%)**	809 (52.57)	640 (46.68)	638 (46.60)	734 (50.10)	
	**Control n (%)**	730 (47.43)	731 (53.32)	731 (53.40)	731 (49.90)	
**Overall**						
Reduced^a^	OR (95% CI)	1.00	0.77 (0.66–0.90)	0.76 (0.66–0.89)	0.89 (0.77–1.03)	0.118
	P*_overall_	0.001				
Complete^b^	OR (95% CI)	1.00	0.79 (0.68–0.93)	0.82 (0.70–0.96)	0.92 (0.78–1.07)	0.334
	P*_overall_	0.017				
**Stratified by smoking status**						
**Never Smokers**						
Reduced^c^	OR (95% CI)	1.00	0.72 (0.51–1.01)	0.72 (0.51–1.02)	0.71 (0.50–1.01)	0.070
	P*_overall_	0.150				
Complete^d^	OR (95% CI)	1.00	0.70 (0.49–0.99)	0.73 (0.51–1.04)	0.68 (0.47–0.98)	0.058
	P*_overall_	0.118				
**Former Smokers**						
Reduced^c^	OR (95% CI)	1.00	0.86 (0.68–1.08)	0.88 (0.70–1.11)	1.02 (0.81–1.28)	0.843
	P*_overall_	0.376				
Complete^d^	OR (95% CI)	1.00	0.93 (0.72–1.19)	0.98 (0.76–1.26)	1.13 (0.88–1.44)	0.299
	P*_overall_	0.471				
**Current Smokers**						
Reduced^c^	OR (95% CI)	1.00	0.71 (0.55–0.91)	0.66 (0.52–0.84)	0.84 (0.67–1.06)	0.113
	P*_overall_	0.004				
Complete^d^	OR (95% CI)	1.00	0.72 (0.55–0.94)	0.73 (0.56–0.95)	0.86 (0.67–1.11)	0.260
	P*_overall_	0.047				

a Adjusted for sex, race/ethnicity, age (continuous), smoking status, total caloric intake.

b Adjusted for sex, race/ethnicity, age, pack-years, total caloric intake, family cancer history, dust exposure, second-hand smoke, emphysema, hay fever, smoking status, asthma, addiction index, alcohol, time since smoking cessation.

c Adjusted for sex, race/ethnicity, age (continuous), total caloric intake.

d Adjusted for sex, race/ethnicity, age, pack-years, total caloric intake, family cancer history, dust exposure, second-hand smoke, emphysema, hay fever, asthma, addiction index, alcohol, time since smoking cessation.

* p-value, chi-square test using quartiles of choline intake as categorical variable.

** p-value, test for linear trend using quartiles of choline intake as continuous variable.

## Discussion

In the present study, we report for the first time the association between choline and betaine dietary intake and lung cancer risk. Our study showed that both choline and betaine dietary intake were significantly associated with lung cancer risk, but evidence for the effect of betaine was stronger. Specifically, compared to those in the lowest quartile of intake, individuals with higher betaine intake had lower odds for lung cancer. A statistically significant trend was observed with increased betaine intake and decreased lung cancer odds, suggesting that higher intake of betaine may be protective against lung cancer. Likewise, a significant protective effect was observed for choline intake, but no linear trend was noted.

Multiple studies have examined the association between choline and betaine dietary intake and various types of cancer, and different effects of the two nutrition factors have been reported. Higher choline intake had been observed to be associated with elevated risk of colorectal adenoma, while increasing betaine intake was associated with reduced risk of the disease [20]. Xu *et al.* reported in a population-based case-control study that higher intake of free choline was associated with lower risk of breast cancer. Higher intakes of betaine, free choline and phosphocholine were associated with reduced all-cause as well as breast cancer-specific mortality, while no association was observed for total choline intake on either the risk or mortality of breast cancer [23], [24]. Reports from existing studies showed more variation in the effect of choline intake compared to betaine intake. This is probably because various sources contribute to total choline intake (free choline, phosphocholine, phosphatidylcholine, etc.), while the intake source for betaine is relatively simple. As a result, it might be more difficult to obtain accurate measurement of total choline intake, which therefore, leads to high variation in the observed associations. It is also possible that, although choline and betaine are in the same metabolic pathway, different mechanisms are involved in the downstream effects in different diseases. Changes in choline metabolism have been reported in many types of cancers. Increased rate of choline transport and elevated phosphorylation of choline to phosphocholine and oxidation to betaine were observed in breast cancer [16]. CHK and PC-specific phospholipase C, enzymes that contribute to PC biosynthesis and degradation, were activated in both epithelial ovarian cancer cell line as well as cancerous tissue [17]. Similarly, elevated levels of CHK, phosphocholine and PC were detected in colon cancer tissue, suggesting that activation of choline metabolic pathways may play a role in carcinogenesis [18], [19].

Cigarette smoking has been observed to increase energy expenditure [36], but the mechanisms remain unknown. Smokers have different dietary preferences than non-smokers. In general, smokers tend to eat less fruits and vegetables than non-smokers, but consume more meat products, saturated fat, coffee, and alcohol, and have higher total energy intake [34], [37]. Consistent with previous studies, we observed a significant increase of total caloric intake with smoking. In addition, a significant increase of choline intake, along with a reduction of betaine intake, was observed from never- to former to current smokers. Since meat, egg yolk, liver, and bacon are major sources of choline, and whole wheat products and green vegetables are high in betaine, our finding that smoking is associated with lower betaine but higher choline intake is reasonable.

We observed a strong influence of smoking on the inverse association between betaine intake and lung cancer. Analyses stratified by smoking status revealed that a higher intake of betaine had no statistically significant association with lung cancer risk among never-smokers. However, higher betaine intake was significantly associated with lower lung cancer risk among both current and former smokers, with a stronger protective effect in current smokers. It is well known that smoking is the strongest risk factor for lung cancer. Our findings suggest that betaine intake may mitigate the adverse effect of smoking, while it may not be relevant as a protective factor among never smokers. Further studies are needed to elucidate the mechanisms of this association.

The effect of smoking on the association between choline intake and lung cancer was less evident compared to betaine. Pack-years of smoking was not significantly associated with choline intake, and no confounding effect of the variable was observed for the association between choline intake and lung cancer. The influence of smoking status on the association between choline intake and lung cancer was also not as obvious as that of betaine intake. A significant association was only present among current smokers, and no linear trend was observed with increased intake. Our results suggest that choline intake may also mitigate the adverse effect of smoking on lung cancer, but the effect was milder, and was independent of the amount of intake.

We observed that both choline and betaine intake were negatively associated with lung cancer risk. Although to our knowledge, no study has been reported on the effect of dietary choline and betaine intake on smoking or lung cancer, multiple studies have revealed strong associations between major nutritional factors in one-carbon metabolism and lung cancer and/or smoking. The results were largely consistent with our findings. Plasma choline and betaine concentrations were reported to be inversely associated with smoking [5]. It has been reported that dietary folate intake was associated with reduced risk of lung cancer among both former [32], [38] and current smokers [38]. A significant inverse association between lung cancer risk and serum concentrations of folate, methionine, and vitamin B6 in former and current smokers was also reported [39]. Choline, methionine and folate interact where homocysteine is converted into methionine [40]. During choline deprivation, the consumption of folate increased in rats [41], while folate deficiency resulted in reduced choline concentrations in liver of rats [42]. Because of the inter-relationships among nutrients involved in one-carbon metabolism, we believe that our findings of a potential protective effect of choline and betaine intake against lung cancer are consistent with previous studies.

One-carbon metabolism includes a complex network of biochemical pathways, which involve interactions between choline, betaine, several group B vitamins, as well as homocysteine and methionine (Figure 1) [40]. Epigenetic variations of DNA, particularly the CpG sites methylation, are important for genome regulation [43]–[45]. Disruption of DNA methylation and impaired DNA repair due to deficiency of methyl donors (folate, choline, betaine or methionine) in one-carbon metabolism were thought to be the underlying mechanism for carcinogenesis [46]–[48]. Given these hypotheses, high intake of choline and betaine may help prevent the adverse effect resulted from hypomethylation of DNA or restore DNA repair mechanism, and therefore, lead to reduced cancer risk.

Alterations in DNA methylation by smoking may contribute to the differential effect of smoking on the association between choline/betaine intake and lung cancer. Smoking has been associated with both altered global methylation [49], [50] and differential methylation in cancer-related genes [51]–[53]. Breitling *et al*. reported a genome-wide study that identified a CpG locus with significant lowered methylation in heavy smokers as compared to never smokers [54]. One can hypothesize that the variation of the effect of betaine by smoking status might due to difference in methylation patterns in smokers and non-smokers. However, our study is not designed to make any mechanistic conclusions, and a separate study should be undertaken to further explore this possibility.

The current study covers a broad range of ages (from 21 to 94) and race/ethnic groups (African Americans, Hispanics, and non-Hispanic Whites). Compared to many other case-control studies, our study included a large number of individuals and sufficient numbers of cases and controls for all smoking and dietary intake categories. Therefore, it allowed stratified analyses with relatively large sample size in each stratum. In addition, since the survey was conducted on newly diagnosed cancer patients, and the patients were asked to report their pre-diagnostic diet (one year prior to diagnosis), any post-diagnostic changes in diet should not affect the results.

Limitations in this case-control study include possible selection bias. Controls in the current study were recruited from clinics. Compared to healthy controls from the general population, these controls were more likely to have other types of diseases, and therefore, the distribution of choline and betaine consumption in our controls may not completely reflect that of controls recruited from general population. However, since MDACC is a major referral center for cancer cases in Houston area, and that Kelsey-Seybold Clinics is the largest private medical group that includes more than 20 clinics throughout Houston, cases and controls are representative of individuals of the same area. Furthermore, unrestrained sample collection by age, sex and ethnicity ensured that conclusions were drawn on a wider and more general population base, which may help alleviate the problem of selection bias. Secondly, due to the nature of survey studies, inaccurate recall may be a problem, and FFQ may introduce measurement errors that lead to biased estimates, likely towards the direction that attenuates the true association [55]. Although it is more likely that the nutritional intake affects the risk of cancer than the reverse, it is also possible that dietary intake is not the direct cause of the disease, but rather confounds the effect of other factors. A cohort study with long-term nutrition intake data or a Mendelian randomization analysis of genetic markers involved in choline and betaine metabolism may help us better understand the relationship between choline and betaine intake and risk of the disease. Moreover, we cannot rule out the possibility of potential residual confounding or unidentified confounders during our analysis, which may lead to biased results.

In conclusion, we observed that both choline and betaine dietary intake were protective against lung cancer, but the protective effect was more evident with betaine intake, and was strongly influenced by smoking. We propose that dietary intake of choline and betaine, especially higher betaine intake, may reduce lung cancer risk by mitigating the adverse effect of smoking.
